# Matrix Metalloproteinases and Stress Hormones in Lung Cancer Progression

**DOI:** 10.1155/2022/5349691

**Published:** 2022-09-29

**Authors:** Georgina Gonzalez-Avila, Bettina Sommer, A. Armando Garcia-Hernandez, Carlos Ramos, Javier Delgado, Lilia Vazquez, Rosa A. Gonzalez, Cuauhtemoc Sandoval, Edgar Flores-Soto

**Affiliations:** ^1^Laboratorio Oncología Biomédica, Instituto Nacional de Enfermedades Respiratorias “Ismael Cosío Villegas”, Calzada de Tlalpan 4502, Col. Sección XVI, Tlalpan, 14080 Ciudad de México, CP, Mexico; ^2^Departamento de Investigación en Hiperreactividad Bronquial, Instituto Nacional de Enfermedades Respiratorias “Ismael Cosío Villegas”, Calzada de Tlalpan 4502, Col. Sección XVI, Tlalpan, 14080 Ciudad de México, CP, Mexico; ^3^Departamento de Investigación en Fibrosis Pulmonar, Instituto Nacional de Enfermedades Respiratorias “Ismael Cosío Villegas”, Calzada de Tlalpan 4502, Col. Sección XVI, Tlalpan, 14080 Ciudad de México, CP, Mexico; ^4^Departamento de Farmacología, Facultad de Medicina, Universidad Nacional Autónoma de México, Coyoacán, 04510 Ciudad de México, CP, Mexico

## Abstract

Several matrix metalloproteinases (MMPs) and psychological stress are associated with poor cancer prognosis. The current work goal was to determine MMPs' and stress hormones' blood concentrations from lung adenocarcinoma (LAC) patients. Patients were divided into the following groups: tobacco smokers (TS), wood smoke-exposed (W), passive smokers (PS), TS exposed to wood smoke (TW), and patients with no recognizable risk factor (N). MMPs, tissue inhibitors of metalloproteinases (TIMPs), adrenaline, noradrenaline, and cortisol blood concentrations were measured by ELISA. Zymography and Western blot assays were performed to determine MMP-2 and MMP-9 active and latent forms. MMP-2, MMP-3, MMP-9, and TIMP-1 blood concentrations, and MMP-9 gelatinase activity were augmented, while MMP-12, MMP-14, and TIMP-2 were diminished in LAC patients. Cortisol was increased in LAC samples. Adrenaline concentrations were higher in W, TS, and TW, and noradrenaline was increased in W and N groups. Positive correlations were observed among cortisol and TIMP-1 (*r*_*s*_ = 0.392) and TIMP-2 (*r*_*s*_ = 0.409) in the W group and between noradrenaline and MMP-2 (*r*_*s*_ = 0.391) in the N group. MMPs' blood concentration increments can be considered as lung cancer progression markers. Although stress hormones were also augmented, only weak correlations were observed between them and MMPs and TIMPs.

## 1. Introduction

Lung cancer incidence reached 2.2 million new cases, and mortality increased to 1.8 million deaths in 2020 worldwide, with metastases being the leading cause of death [1, 2]. In this context, one of the skills that tumor cells acquire to spread to other localizations is the increase of matrix metalloproteinases' (MMPs) expression [3]. MMPs are a group of zinc and calcium-dependent endopeptidases that belong to the metzincin family, which includes meprins, a disintegrin and metalloproteinases (ADAMs), a disintegrin and metalloproteinases with thrombospondin motifs (ADAMTs), reprolysins, serralysins, and astacins [4]. Up to date, 24 MMPs have been recognized in humans and cataloged according to their molecular structure and substrate specificity in gelatinases, matrilysins, collagenases, stromelysins, glycosylphosphatidylinositol-anchored (GPI-anchored) MMPs, transmembrane-type I MMPs, transmembrane-type II MMPs, and other MMPs [3, 5].

MMPs' expression can be conditioned by cytokines, hormones, ultraviolet (UV) irradiation, oncogene products, growth factors, retinoic acids, interleukins, cell-cell interactions, and extracellular matrix (ECM)-cell interactions, as well as changes in the extracellular environment like acidification and hypoxia as it occurs in the tumor microenvironment (TME) [3, 6].

Likewise, the MMPs' enzymatic activity is explicitly regulated by the tissue inhibitors of metalloproteinases (TIMPs) which block MMPs' activity by establishing a tight 1 : 1 stoichiometric complex between their N-terminal motif and the catalytic site of MMPs [7]. Four different TIMPs have been identified: (1) TIMP-1, a 28 kDa glycoprotein; (2) TIMP-2, a 21 kDa nonglycosylated protein; (3) TIMP-3, a 24/27 kDa glycosylated protein attached to the extracellular matrix; and (4) TIMP-4, a 22 kDa nonglycosylated protein. The TIMP family has an essential role in cancer progression, participating in events such as pericellular proteolytic activity during the invasion process and the control of angiogenesis, protecting the integrity of new blood vessels [7, 8].

MMPs participate throughout the whole metastatic cascade due to their capacity to modify the ECM and basement membranes and activate and release chemokines, growth factors, cytokines, adhesion, and cytoskeletal molecules [9]. The increase of several MMPs' expression and enzymatic activity, such as MMP-1, MMP-2, MMP-3, MMP-7, MMP-9, MMP-10, and MMP-14, correlates with the aggressiveness of different types of cancer, which leads to consider them as prognostic markers and targets of new therapeutic strategies [10]. Likewise, it is worth remarking that some MMPs have protective effects against cancer progression, for instance, MMP-12 [3].

On the other hand, chronic stress and depression have been linked to cancer onset, progression, and mortality [11, 12]. Furthermore, when patients suspect the presence of cancer, they become stressed, a psychological state that increases once the diagnosis is confirmed and during the whole evolution of the disease [13]. A physiological stress response includes the stimulation of the sympathetic nervous system and the hypothalamic-pituitary-adrenal (HPA) axis that triggers the secretion of catecholamines and glucocorticoids that can arrive at the tumor via blood circulation [12]. Moreover, neurotransmitters such as adrenaline and noradrenaline are released directly at the tumor tissue by sensory and autonomic nerves from the own tumor innervation [14].

*In vitro* and animal model experiments have demonstrated that stress hormones interfere with the immune surveillance and promote tumor cell proliferation, resistance to apoptosis, epithelial-mesenchymal transition (EMT), angiogenesis, and invasion, including MMPs' production [15]. However, little information exists regarding the effects of stress hormones on the MMPs' and TIMPs' synthesis in humans. Therefore, the purpose of the current work was to determine if a rise in MMPs', TIMPs', and stress hormones' concentrations exists in blood samples from lung adenocarcinoma patients with advanced stages of the disease. Likewise, we examined whether there was a correlation between MMPs' and TIMPs' blood concentrations with elevated levels of adrenaline, noradrenaline, or cortisol.

## 2. Materials and Methods

### 2.1. Lung Cancer Patients

One hundred and four primary lung adenocarcinoma (LAC) subjects, 47 females, age 57.7 ± 11.9, and 57 males, age 61.9 ± 10.7 years, fulfilled the criteria for the present research. The histological diagnosis was determined by examining tissue samples obtained by percutaneous needle biopsy or bronchoscopy. The stage of the disease was established before treatment, according to the TNM classification [16]. Patients were distributed in groups depending on the risk factors associated with lung cancer onset: (1) LAC associated with wood smoke exposure (W) included 38 never-smoker patients with an indoor wood smoke exposure of 30.5 ± 3.4 years (170 ± 30 h/year; range, 8 to 720 h/year); (2) LAC associated with tobacco smoking (TS) consisted of 22 patients who were current smokers for >10 years (33.1 ± 3.8 years) with a mean smoking history of 27.29 ± 5.4 pack-years (range, 1.6 to 80 pack-years); (3) a negative group (N) formed by 26 patients with LAC with no history of tobacco smoking, radon, wood smoke, asbestos, or solvent exposure, nor a family history of cancer; (4) LAC associated with passive smoking (PS) consisted of 10 patients that were no tobacco smokers, but had a history of tobacco smoke exposure of 21.6 ± 5.6 years (154 ± 57.7 h/year; range, 8 to 400 h/year); and (5) LAC associated with tobacco smoking and wood smoke exposure (TW) comprised of 8 patients who were current smokers for 40.6 ± 7.3 years, with a mean smoking history of 23.8 ± 7.8 pack-years (range, 5 to 48 pack-years), and a history of wood smoke exposure of 174.5 ± 69.7 h/year (range, 20 to 592 h/year) for 32.6 ± 10.5 years. The control group included 100 healthy never-smoker volunteers (49 females, age 53.4 ± 7.04, and 51 males, age 56.3 ± 10.7 years), without wood or tobacco smoke exposure, with normal spirometry values, no history of asthma, allergy, or atopy, and without signs of infectious respiratory diseases.

Written informed consent was provided from volunteer subjects and lung cancer patients. The Institutional Ethical and Research Committees authorized the study, and it was conducted under the amended Declaration of Helsinki. Blood samples were obtained from healthy subjects and LAC patients before starting cancer treatment in the morning between 8 : 00 and 9 : 00 AM, to avoid cortisol blood concentration differences due to circadian rhythmicity. Blood samples (liquid biopsy) were chosen for this study since the method to get them is not as invasive as tissue biopsies used for diagnostic purposes. Moreover, the tissue samples obtained by biopsy were not enough to complete all the experimental tests.

Protein content from plasma and serum was assessed by the bicinchoninic acid protein method (Pierce Chemical Co, Rockford, III, USA) [17].

### 2.2. Quantification of MMPs, TIMPs, and Stress Hormones

MMPs', TIMPs', cortisol, adrenaline, and noradrenaline concentrations from plasma or serum samples were determined by the ELISA technique following the manufacturers' instructions. MMP-2, MMP-3, TIMP-1, and TIMP-2 quantities were measured with Quantikine Human R&D Systems ELISA kits (R&D Systems, Minneapolis, MN, USA), and the minimum detectable doses (MDDs) for them were 0.016 ng/ml, 0.002 ng/ml, 0.08 ng/ml, and 0.004 ng/ml, respectively. MMP-9 and MMP-12 plasma amounts were quantified with OriGene Technologies ELISA kits (OriGene Technologies, Inc., Rockville, MD, USA), and their MDDs were 3 pg/ml and 10 pg/ml, respectively. MMP-14 and cortisol concentrations were measured with Abcam ELISA kits (Abcam, Cambridge, MA, USA), and their MDDs were 0.145 ng/ml and 2.44 ng/ml, respectively. Adrenaline and noradrenaline quantifications were done with BI-CAT ELISA kit from Eagle Biosciences kits (Eagle Biosciences, Inc., Nashua, NH, USA), and their MDDs were 5 pg/ml and 16 pg/ml, respectively.

### 2.3. Carcino-Embryonic Antigen (CEA) Quantification

CEA plasma concentrations were quantified by chemoluminescent microplate immunoassay (CMIA) (Abbott, Laboratories, Abbott Park, IL, USA) following the manufacturers' instructions. According to the kit's instructions, the CEA MDD was better than 0.5 ng/ml at the 95% level of confidence.

### 2.4. Gelatin Zymography Assay

MMPs' gelatinolytic activity was analyzed using 8% SDS-PAGE gels containing 0.01% gelatin from porcine skin (Sigma-Aldrich Co, St. Louis, MO, USA). Thirty micrograms of serum protein from each subject were added per lane under nonreducing conditions, and electrophoresis was run under constant current (10 mA). Gels were rinsed in 2.5% Triton X-100 and subsequently incubated in 50 mmol/L Tris-HCl buffer, containing 20 mmol/L CaCl_2_ and 5 mmol/L ZnCl_2_, pH 7.4, at 37°C for 24 h. Then, they were stained with 0.05% Coomassie blue G-250 (BioRad, Richmond, Calif, USA), and the gelatinase activity was seen as transparent bands against a blue background on the dyed gels.

### 2.5. Western Blot Analysis

Western blot was carried out on 8% SDS-PAGE gels to identify which gelatinase corresponds to the lytic bands observed in the zymography assay. Thirty micrograms of serum protein from representative control and cancer subjects' samples used in the zymography technique were applied per lane after sample reduction with 5% *β*-mercaptoethanol boiled at 100°C for 10 min. Following electrophoresis, proteins were transferred to PVDF membranes. Membranes were then blocked with 2.5% nonfat dry milk in TTBS buffer (100 mM Tris-HCl, pH 7.5 buffer containing 0.1% Tween 20 and 150 mM NaCl) and incubated for 90 min with 10 *μ*g/ml anti-MMP-2 mouse monoclonal antibody (Abcam) or 7 *μ*g/ml anti-MMP-9 mouse monoclonal antibody (Biolegend, San Diego, CA, USA). Anti-beta-tubulin (1 : 200) (Abcam) was used as the loading control. Bands were detected with the VECTASTAIN ABC kit (Vector Laboratories, Burlingame, CA, USA).

### 2.6. Statistical Analysis

Mann–Whitney *U* test was performed to analyze data, and values were expressed as mean ± standard error (SE) of the mean for at least three assays. Comparisons among groups were made using the Kruskal-Wallis ANOVA analysis. Spearman correlation coefficient (*r*_*s*_) and linear regression (*R*^2^) were employed to determine correlations among groups. Statistical significance was considered as *p* < 0.05 for all results. The SPSS version 20.0 statistical software (IBM Corp., Armonk, NY) was utilized for all statistical analyses.

## 3. Results

### 3.1. Primary Lung Adenocarcinoma Patients' Clinical Profile

In this work, of 104 patients studied (100%), 71.2% (74/104) were never-smokers; from the 74 never-smoker subjects (100%), 51.4% (38/74) belonged to the W group, 35.1% (26/74) to the N group, and 13.5% (10/74) to the PS group (Table 1). The W group was constituted mainly of women (63.2%, 24/38), while men comprised 36.8% (14/38). The N group consisted of 53.8% (14/26) men and 46.2% (12/26) women. In men, the principal cause of lung cancer was still associated with tobacco smoking (36.8%, 21/57); however, considering the number of men in the W and N groups (*W* = 14/57, and *N* = 14/57), we observed more men in the never-smoker (28/57, 49.1%) than in the TS group. Surprisingly, we only detected one woman with a history of tobacco smoking in our studied population. Passive smoking, as a risk factor for lung cancer, was found in 7 women and 3 men, and the combination of tobacco smoking and wood smoke exposure was observed in 5 men and 3 women. Likewise, most LAC patients were diagnosed with advanced stages of the disease (IV =90.4%).

Additionally, the original study design for this work was to examine LAC patients and healthy subjects. However, when our lung cancer patients' clinical characteristics were revised, distinct associated risk factors were detected, and therefore, patients were divided into groups according to this observation to broaden further the scope of the present research. Consequently, results from the whole LAC population and each group are reported separately.

### 3.2. CEA Plasma Concentrationas in Advanced Stages of Lung Adenocarcinoma

The CEA was quantified in plasma samples from lung cancer patients as another clinical method to evaluate cancer progression. In the patients studied in the present work, CEA was measured but only in 70 of 104 (70/104, 67.31%) patients obtaining values of 62.5 ± 14.9 ng/ml with a range from 0.5 to 626.43 ng/ml (Table 2). Thirty (30/70, 42.86%) had low CEA concentrations (2.3 ± 0.3 ng/ml) with a range from 0.5 to 4.92 ng/ml which are between the normal reference values (0 to 5 ng/ml). In addition, 40 (40/70, 57.14%,) had a value higher than the reference value (107.7 ± 23.8 ng/ml) with a range from 6.01 to 626.43 ng/ml. Interestingly, significant differences were observed between the W (49.5 ± 16.9 ng/ml) and PS (13.1 ± 10.5 ng/ml) groups (*p* = 0.02).

Because CEA was not measured in all cases and variation in its concentrations varied so much, it could not be considered as a reliable biomarker in this study.

### 3.3. MMP-2, MMP-3, and MMP-9 Amounts Were Increased in Lung Cancer Patients

**Figure 1:**
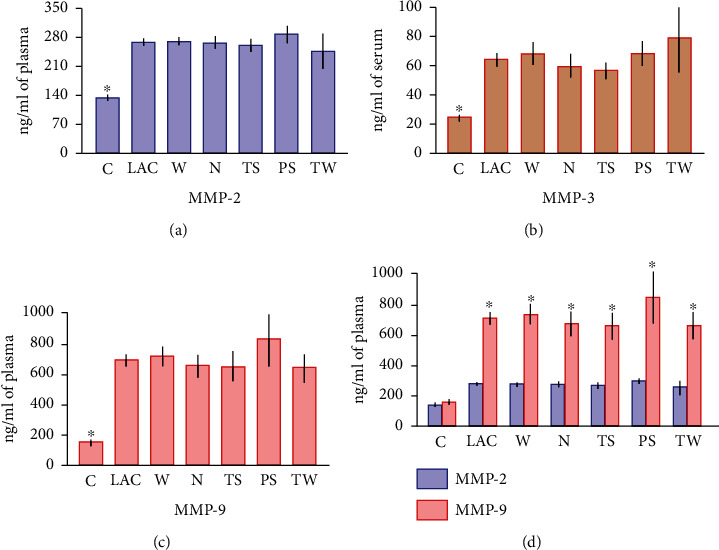
MMPs' concentrations in blood samples from lung adenocarcinoma patients. MMPs were quantified in plasma or serum samples from control and LAC subjects by ELISA technique. (a, b, c) MMP-2, MMP-3, and MMP-9 were significantly enhanced in LAC samples compared to the control group (^∗^*p* < 0.0001). No significant differences were identified among lung cancer groups. (d) MMP-9 concentrations were significantly increased compared to MMP-2 levels in all cancer groups (^∗^*p* < 0.0001). No significant differences were detected between MMP-2 and MMP-9 in control subjects. Bars represent the mean ± standard error of the mean. Abbreviations: MMP: matrix metalloproteinase; C: control group; LAC: lung adenocarcinoma; W: LAC associated with wood smoke exposure; N: LAC patients with no recognizable risk factor; TS: LAC associated with tobacco smoking; PS: LAC associated with passive smoking; TW: LAC associated with tobacco smoking and wood smoke exposure.

MMP-2 plasma concentrations were significantly augmented in the LAC group (all lung cancer patients examined) when compared to healthy subjects (266.7 ± 6.3 ng/ml and 133.2 ± 4.2 ng/ml, respectively; *p* < 0.0001) (Figure 1(a)). Although the highest concentration of MMP-2 was observed in the PS group (286.9 ± 17.5 ng/ml), differences among groups were not significant. Likewise, MMP-3 amounts were significantly elevated in LAC patients in comparison with the control group (64.6 ± 4.2 ng/ml and 24.6 ± 1.3 ng/ml, respectively; *p* < 0.0001) (Figure 1(b)). The highest MMP-3 levels were found in the TW group (79.1 ± 23.9 ng/ml), but differences between the groups were not significant. Similarly, MMP-9 concentrations were also significantly increased in the LAC group in comparison to control subjects (691.8 ± 36.5 ng/ml and 152.7 ± 12.9 ng/ml, respectively, *p* < 0.0001) (Figure 1(c)). The highest MMP-9 amount was identified in PS patients (827.5 ± 165.5 ng/ml), but differences among groups were not significant. Interestingly, concentrations of MMP-9 were higher than MMP-2 levels (*p* < 0.0001) when both gelatinases were compared in cancer groups. In contrast, differences among these MMPs' concentrations were not significant in samples from control subjects (Figure 1(d)).

### 3.4. Latent and Active MMP-2 and MMP-9 Forms with Gelatinase Activity in Serum from Lung Cancer Patients

**Figure 2:**
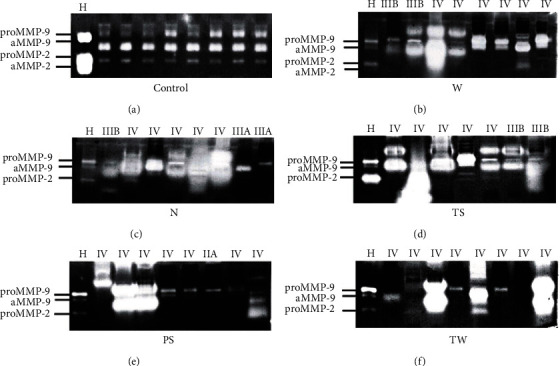
MMP-2 and MMP-9 gelatinase activity in serum from lung cancer patients. Zymography assay showed a rise in the gelatinase activity of MMP-2 and MMP-9 in serum from most lung cancer patients, particularly in the W (b), N (c), and TS (d) groups. Disease stages are indicated at the top of each gel. (a) Control group; (b) LAC associated with wood smoke exposure, W group; (c) LAC patients with no recognizable risk factor, N group; (d) LAC associated with tobacco smoking, TS group; (e) LAC associated with passive smoking, PS group; (f) LAC associated with tobacco smoking and wood smoke exposure, TW group. Abbreviations: LAC: lung adenocarcinoma; H: HT-1080 fibrosarcoma cell medium used as an enzymatic activity control; aMMP-2: active MMP-2; aMMP-9: active MMP-9; pMMP-9: pro-MMP-9 (latent enzyme).

The zymography assay revealed the presence of different molecular forms of MMP-2 and MMP-9 with the capacity to degrade gelatin; the activity of both enzymes was augmented in most lung cancer patients (Figures 2(b)–2(f)) in comparison with control samples (Figure 2(a)). Notably, heterogeneous gelatinase activity was observed in patients from the PS and TW groups (Figures 2(e) and 2(d)), regardless of the disease stage. Likewise, low gelatinase activity corresponding to proMMP-9 was observed in N and PS groups (Figures 2(c) and 2(e)) in patients with stages IIIA and IIA, respectively. Moreover, gelatinase activity from MMP-2 was not detected, particularly in some samples from PS and TW groups (Figures 2(e) and 2(f)). Similarly, MMP-9 gelatinase activity was increased in IIIB and IV stages in W, N, and TS groups (Figures 2(b)–2(d)). High molecular weight bands with gelatinase activity were also observed. These bands may correspond to MMP-9 polymers or complexes among MMP-9 and neutrophil gelatinase B-lipocalin (NGAL) [18]. It is important to consider that control and cancer samples were run without a prepurification step, and therefore, the mobility of the lytic bands is slower due to the interactions of MMPs with other proteins present in the serum [18].

**Figure 3:**
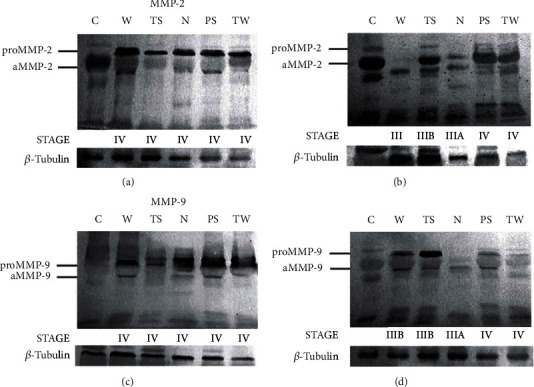
MMP-2 and MMP-9 immunoblotting. (a) Bands corresponding to proMMP-2 and active MMP-2 were detected by Western blot in serum from healthy and lung cancer patients with disease stage IV. (b) ProMMP-2 bands' intensity was lower in samples from patients with stages IIIA, IIIB, and some stage IV. Active MMP-2 bands were thinner in patients from N and W groups with stages IIIA and IIIB, respectively. (c) MMP-9 active and latent forms were detected in LAC patients with stage IV. (d) Some stage IIIA and IV patients' samples showed lower intensity in bands corresponding to MMP-9 latent and active forms. Disease stages are indicated at the bottom of each blot. Abbreviations: MMP: matrix metalloproteinase; C: control group; LAC: lung adenocarcinoma; W: LAC associated with wood smoke exposure; N: LAC patients with no recognizable risk factor; TS: LAC associated with tobacco smoking; PS: LAC associated with passive smoking; TW: LAC associated with tobacco smoking and wood smoke exposure.

Western blot analysis was performed to identify MMP-2 and MMP-9 active and latent forms in LAC patients (Figure 3). MMP-2 immunoblotting showed bands corresponding to proMMP-2 and this enzyme's active form (Figures 3(a) and 3(b)). The proMMP-2 band density was higher in LAC patients with stage IV of the disease than in the control subjects (Figure 3(a)). In contrast, low density bands corresponding to proMMP-2 were observed in samples from patients with stages IIIA and IIIB and some subjects with stage IV (Figure 3(b)). Bands corresponding to the MMP-2 active form were also identified in control and cancer patients. This band had a similar intensity in control and patients with the disease at stage IV (Figure 3(a)) even though it was thinner in samples from patients with stages IIIA and IIIB from N and W groups, respectively (Figure 3(b)). In addition, lower bands observed at the bottom of the blot may correspond to MMP-2 fragments.

MMP-9 Western blot assay detected bands that correspond to proMMP-9 and aMMP-9; interestingly, these bands showed high intensity in samples from cancer patients at stage IV compared to control subjects (Figure 3(c)). Both MMP-9 forms were identified with lower intensity in a patient from the N group at stage IIIA, while bands corresponding to some patients with stage IV from PS and TW groups had densities similar to the bands corresponding to control subjects' samples (Figure 3(d)). Bands with higher molecular weights were also observed. These bands may correspond to MMP-9 polymers and/or complexes of MMP-9 with other molecules such as NGAL. MMP-9 fragments with low molecular weights were also detected. Western blot results paralleled those obtained in the zymography analysis. The mobility of the bands might have been affected by the interaction of MMPs with other serum proteins.

### 3.5. MMP-12 and MMP-14 Concentrations Were Decreased in Lung Adenocarcinoma

**Figure 4:**
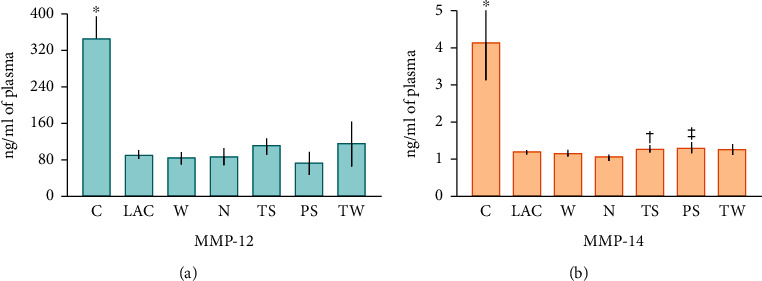
MMP-12 and MMP-14 plasma concentrations. MMP-12 (a) and MMP-14 (b) were significantly decreased in LAC patients compared to healthy subjects (^∗^*p* < 0.0001). There were no significant differences among cancer groups in MMP-12 concentrations. Contrastingly, TS and PS groups showed higher MMP-14 concentrations than the N group (†*p* = 0.03 and ‡*p* = 0.04, respectively). Bars represent the mean ± standard error of the mean. Abbreviations: MMP: matrix metalloproteinase; C: control group; LAC: lung adenocarcinoma; W: LAC associated with wood smoke exposure; N: LAC patients with no recognizable risk factor; TS: LAC associated with tobacco smoking; PS: LAC associated with passive smoking; TW: LAC associated with tobacco smoking and wood smoke exposure.

MMP-12 levels were significantly decreased in samples from LAC patients in comparison with the control group (91.1 ± 7.9 ng/ml and 345.2 ± 48.5 ng/ml, respectively; *p* < 0.0001) (Figure 4(a)). There were no significant differences among lung cancer groups. Likewise, MMP-14 amounts were lower in the LAC group than in healthy subjects (1.2 ± 0.04 ng/ml and 4.1 ± 0.09 ng/ml, respectively; *p* < 0.0001) (Figure 4(b)). The highest concentrations were detected in the TS, PS, and TW groups, but significant differences were only detected among the TS and N groups (1.3 ± 0.08 and 1.05 + 0.05 ng/ml, respectively; *p* = 0.03) and between PS (1.3 + 0.1 ng/ml) and N patients (*p* = 0.04).

### 3.6. TIMP-1 and TIMP-2 Blood Levels

**Figure 5:**
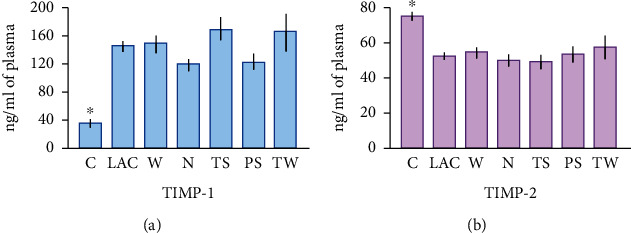
TIMP levels in plasma from lung cancer patients. (a) ELISA results revealed an increase in TIMP-1 in all cancer samples compared to control subjects (^∗^*p* < 0.0001). TIMP-1 concentrations were higher in the TS than in the N group (†*p* = 0.03). (b) TIMP-2 amounts were higher in the C group than in all groups of lung cancer patients (^∗^*p* < 0.0001). No significant differences between lung cancer groups were detected. Bars represent the mean ± standard error of the mean. Abbreviations: TIMP: tissue inhibitor of metalloproteinases; C: control group; LAC: lung adenocarcinoma; W: LAC associated with wood smoke exposure; N: LAC patients with no recognizable risk factor; TS: LAC associated with tobacco smoking; PS: LAC associated with passive smoking; TW: LAC associated with tobacco smoking and wood smoke exposure.

TIMP-1 concentrations were augmented in LAC patients in comparison with control subjects (145.3 ± 6.4 ng/ml and 34.4 ± 2.3 ng/ml, respectively; *p* < 0.0001) (Figure 5(a)). Significant differences were found between TS and N groups (169.1 ± 15.2 ng/ml and 119.4 ± 6.8 ng/ml, respectively; *p* = 0.03) but not among other cancer groups. Likewise, TIMP-2 plasma amounts were lower in LAC patients in comparison to control subjects (52.3 ± 1.5 ng/ml and 75.14 ± 1.5 ng/ml, respectively; *p* < 0.0001) (Figure 5(b)). There were no significant differences among cancer groups.

### 3.7. Stress Hormones' Concentrations Increased in Lung Cancer Patients

**Figure 6:**
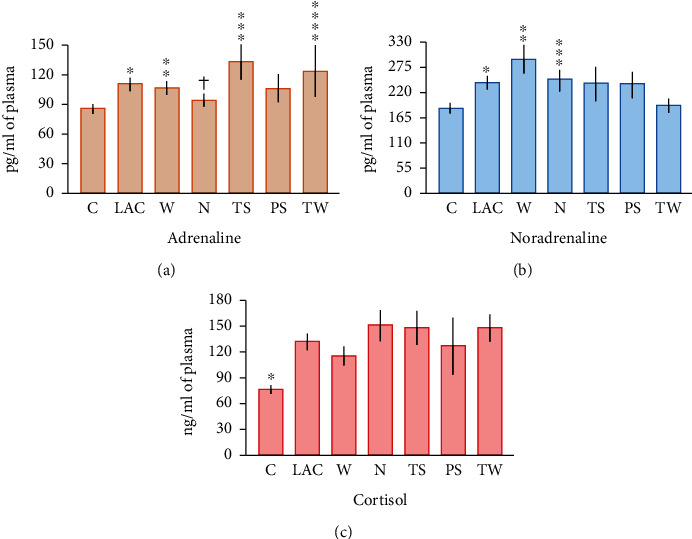
Stress hormones' levels in plasma from lung cancer patients. (a) Adrenaline levels were significantly enhanced in LAC patients compared to healthy subjects (^∗^*p* = 0.0001); however, these differences were only due to the W (^∗∗^*p* = 0.001), TS (^∗∗∗^*p* < 0.0001), and TW (^∗∗∗∗^*p* = 0.005) groups. Significant differences were also found among the TS and N groups (†*p* = 0.03). (b) Noradrenaline was increased in the LAC group compared to control subjects (^∗^*p* < 0.01). These differences were due to the W (^∗∗^*p* = 0.003) and N (^∗∗∗^*p* = 0.02) groups when compared to control subjects. (c) Cortisol concentration was significantly augmented in all cancer patients compared to healthy subjects (^∗^*p* < 0.0001). Bars represent mean ± standard error. Abbreviations: C: control group; LAC: lung adenocarcinoma; W: LAC associated with wood smoke exposure; N: LAC patients with no recognizable risk factor; TS: LAC associated with tobacco smoking; PS: LAC associated with passive smoking; TW: LAC associated with tobacco smoking and wood smoke exposure.

Adrenaline plasma amounts were augmented in the LAC group when compared with control subjects (110.5 ± 5.2 pg/ml and 86.2 ± 3.1 pg/ml, respectively; *p* = 0.0001), but these differences were due to W (106.8 ± 6 pg/ml; *p* = 0.001), TS (133 ± 17.4 pg/ml; *p* < 0.0001), and TW (123.6 ± 26.6 pg/ml; *p* = 0.005) groups (Figure 6(a)). There were no significant differences among adrenaline concentrations from the N and PS groups when compared to the healthy subjects. Furthermore, adrenaline concentrations were significantly lower in N patients (94.4 ± 6.2 pg/ml) compared to TS group (*p* = 0.03). There were no significant differences among other cancer subjects. Similarly, noradrenaline concentrations were increased in the LAC group compared to control subjects (241 ± 12.6 pg/ml and 185.5 ± 9.1 pg/ml, respectively; *p* < 0.01). However, significantly higher noradrenaline levels were only detected in plasma from W (291.5 ± 29.6 pg/ml; *p* = 0.003), and N (247.5 ± 20.8 pg/ml; *p* = 0.02) groups, but not with the other cancer patients when compared to healthy subjects (Figure 6(b)). There were no significant differences between cancer groups. Likewise, cortisol concentrations were enhanced in the LAC group in comparison to control subjects (132.9 ± 8.1 ng/ml and 76.9 ± 4.3 ng/ml, respectively; *p* < 0.0001) (Figure 6(c)). Moreover, cortisol levels were significantly increased in each cancer group compared to control subjects. The highest concentrations were found in the N, TS, and TW groups (151.2 ± 17.4 ng/ml, 148.3 ± 18.4 ng/ml, and 148.3 ± 15.4 ng/ml, respectively), and the lowest levels were observed in W and PS patients (115.3 ± 10.7 ng/ml and 126.6 ± 32.2 ng/ml, respectively). Differences among groups were not significant.

Interestingly, no statistical differences were observed in MMPs' enzymatic activity, MMPs', TIMPs', and stress hormones' concentrations between blood samples from women and men.

### 3.8. Correlations between Blood Concentrations of Stress Hormones with MMPs and TIMPs

**Figure 7:**
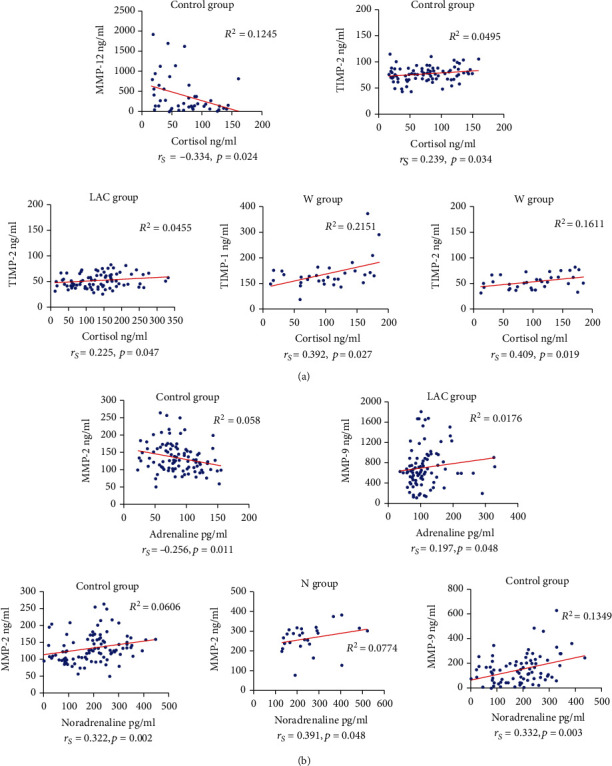
Correlations among stress hormones with MMPs' and TIMPs' blood concentrations. (a) Cortisol correlations: an inverse correlation between cortisol and MMP-12 (*p* = 0.024) and a positive correlation among cortisol and TIMP-2 (*p* = 0.034) were observed in control group samples. Significant positive correlations were also found among cortisol and TIMP-2 in the LAC group (*p* = 0.047) and between cortisol and both TIMPs in samples from W patients (TIMP-1, *p* = 0.019; TIMP-2, *p* = 0.027). *R*^2^ values were low in all examined groups. (b) Catecholamines' correlations: a significant negative correlation (*p* = 0.011) was found between adrenaline and MMP-2 in control subjects. A significant positive correlation (*p* = 0.048) was identified between adrenaline and MMP-9 in the LAC group. Significant positive correlations were found among noradrenaline and MMP-2 (*p* = 0.002) and MMP-9 (*p* = 0.003) in control subjects, and between noradrenaline and MMP-2 in samples from N patients (*p* = 0.048). *R*^2^ values among all the variables analyzed were low. Abbreviations: LAC: lung adenocarcinoma; W: LAC associated with wood smoke exposure; N: LAC patients with no recognizable risk factor; *r*_*s*_, Spearman correlation coefficient.

Spearman correlation analysis was done only for control, LAC, W, N, and TS groups since these groups had enough individuals to carry out the statistical analysis. A significant negative correlation (*r*_*s*_ = −0.334) was found among cortisol and MMP-12 (*p* = 0.024), while a significant positive correlation was determined between cortisol and TIMP-2 (*r*_*s*_ = 0.239, *p* = 0.034) in control subjects. *R*^2^ from linear regression analysis showed a poor association between cortisol and MMP-12 and TIMP-2 (R^2^=0.1245 and *R*^2^ = 0.0495, respectively). Dispersion graphics and linear regressions are shown in Figure 7(a). In addition, significant positive correlations were established among cortisol and TIMP-2 (*r*_*s*_ = 0.225, *p* = 0.047) in the LAC group with an *R*^2^ = 0.0455, and among cortisol and TIMP-1 (*r*_*s*_ = 0.409, *p* = 0.019) and TIMP-2 (*r*_*s*_ = 0.392; *p* = 0.027) in W patients, with low *R*^2^ values (*R*^2^ = 0.2151 and *R*^2^ = 0.1611, respectively) (Figure 7(a)). Other correlations between cortisol and MMPs or TIMPs were not significant. Similarly, a significant negative correlation was obtained among adrenaline and MMP-2 in the control group (*r*_*s*_ = −0.256, *p* = 0.011) with an *R*^2^ = 0.058. A positive correlation was observed among adrenaline and MMP-9 in the LAC group (*r*_*s*_ = 0.197, *p* = 0.048) with a low *R*^2^ value (*R*^2^ = 0.0176). There were no other significant correlations between adrenaline and MMPs or TIMPs in any group (Figure 7(b)). Likewise, significant positive correlations between noradrenaline and MMP-2 and MMP-9 were observed in the control group (*r*_*s*_ = 0.322, *p* = 0.002 and *r*_*s*_ = 0.332, *p* = 0.003, respectively), as well as among noradrenaline and MMP-2 in the N group (*r*_*s*_ = 0.391, *p* = 0.048) (Figure 7(b)). A low correlation among noradrenaline and MMP-2 and MMP-9 (*R*^2^ = 0.0606 and *R*^2^ = 0.1349, respectively) was observed in the control group and between noradrenaline and MMP-2 in the N group (*r*_*s*_ = 0.391, *p* = 0.048, *R*^2^ = 0.0774) (Figure 7(b)).

## 4. Discussion

Lung cancer incidence is still high due, at least in part, to the diverse risk factors involved in its pathogenesis besides tobacco smoking, such as biomass smoke, tobacco smoke (passive smoking), radon, X-rays, and air pollution exposition, preexisting lung disease, and genetic susceptibility [19–23]. In the present study, 71.2% of the lung cancer subjects examined were never smokers, including men and women, and the most frequent risk factor related to their disease was wood smoke exposure. Interestingly, there were patients with no recognizable risk factor (N group). Further research related to this kind of patient is needed.

As already mentioned, in most cases, lung cancer is diagnosed in advanced stages when patients present systemic signs, for instance, weight loss, fatigue, night sweats, and fever, and symptoms caused by intrathoracic spread, paraneoplastic syndromes, endobronchial growth, and distant metastasis [24]. Then, the histological confirmation is performed together with the identification of the histological type. The extension of the disease is categorized in accordance to the TNM classification employing imaging techniques such as positron emission tomography (PET), computed tomography (CT), magnetic resonance imaging (MRI), and single photon emission computed tomography (SPECT); these techniques are also used for monitoring the response to treatment and the evolution of the disease [16, 24]. Moreover, blood sampling (liquid biopsy) has recently been used as a noninvasive technique as part of *in vitro* diagnosis (IVD), allowing the exploration of several molecular markers during the disease evolution [25, 26]. In this context, circulation tumor cells (CTC) and circulating tumor DNA (ctDNA) quantification have been employed to evaluate treatment response and cancer progression, but despite the great specificity of these assays, they have a low sensitivity [27]. In addition, the assessment of blood circulating proteins like CEA, CYFRA 21-1 (serum cytokeratin 19 fragment), fibrinogen, C-reactive protein (CRP), and neuron-specific enolase (NSE), as markers of lung cancer progression, has been considered inconclusive [28]. In the present work, MMPs and TIMPs blood concentrations were quantified in samples from lung adenocarcinoma patients with advanced stages of the disease according to the TNM classification and compared with healthy subjects to identify differences in their levels that may be considered prognostic markers. In this regard, evidence shows that concentrations of MMPs from serum and plasma may be used as diagnostic and prognostic markers in lung cancer. For instance, a rise in serum MMP-2 levels has been identified in advanced stages of nonsmall cell lung cancer (NSCLC) compared with nonmetastatic lung cancer and control subjects [29–31]. Additionally, a decline in MMP-2 and MMP-9 enzymatic activity was found in patients who responded to first-line chemotherapy treatment in comparison to those with a disease progression [32]. Interestingly, patients with an improved response to therapy were never smokers.

In the current work, we found an increment of MMP-2 and MMP-9 levels and their potential enzymatic activity in samples from cancer patients. Both MMPs play an important role throughout the metastatic process [3]. Likewise, MMP-3 was also enhanced in LAC patients compared to healthy subjects. MMP-3 participates actively in cancer progression since it inhibits the Wnt5b favoring the activation of the canonical Wnt signaling pathway to induce cancer stem cells differentiation and expansion [33]. Moreover, MMP-3 disrupts E-cadherin and stimulates *β*-catenin nuclear translocation through Wnt1, promoting the EMT process [34, 35]. MMP-3 also contributes to the activation of proMMP-9, forming the proMMP-9/TIMP-1/MMP-3 activation complex [36]. In contrast, MMP-12 concentrations were lower in cancer patients than in control subjects. In this regard, the evidence suggests that MMP-12 has protective effects in the early stages of NSCLC due to its capacity to inhibit angiogenesis [37]. Conversely, our study population had advanced stages of the disease with low MMP-12 concentrations, which could facilitate cancer progression. Likewise, a decrease in MMP-14 levels was observed in cancer samples compared to the control group. MMP-14 is a membrane-type MMP (MT1-MMP) involved in proMMP-2 activation forming the proMMP-2/TIMP-2/MMP-14 activation complex [38]. Once MMP-2 is active, it is released, while MMP-14 and TIMP-2 remain attached to the membrane; this event may explain the low levels of MMP-14 and TIMP-2 found in blood from lung cancer patients. Additionally, TIMPs' concentrations were also different among control and lung cancer groups. Interestingly, while TIMP-1 plasma levels were high, TIMP-2 amounts were low in all cancer subjects and these differences could be due to the characteristic activation mechanism of proMMP-2 and proMMP-9 that includes the participation of TIMP-2 and TIMP-1, respectively [36, 38].

Noteworthy, the measurement of MMPs and TIMPs in blood samples as part of the IVD can be upgraded using nanotechnology, that is, coupling nanomaterials to different techniques such as electrochemical and optical biosensors [26]. In this regard, a biosensor formed by photoluminescent quantum dots (QDs) with a CdSe/ZnS core/shell structure, conjugated with biotin and the GPLGVRGK peptide, functionalized with streptavidin and a black hole quencher (BHQ), has been constructed to quantified MMP-2 [39]. This system can be improved by employing InP instead of Cd in the QDs structure to decrease its toxicity [40]. Furthermore, the construction of InP/ZnSe/ZnS//ZnSQDs probes has proven to give the system major stability in aqueous solutions. In addition, MMPs can be directly visualized and targeted in lung cancer tissue with the use of nanotheranostic platforms conjugated with peptides degradable by these enzymes [10]. Moreover, nanoprobes containing MMPs' detection systems can be coated with cell membranes, for instance, red blood cell membranes or cancer cell membranes (CCMs), to avoid the clearance of nanocarriers by the reticuloendothelial system (RES) [41]. Additionally, the fusion of CCMs with other types of cell membranes (engineered CCMs [ECCMs]) takes advantage of their characteristics, allowing its use for theranostic purposes.

On the other hand, studies done in vitro and in vivo point out that chronic stress favors cancer progression by promoting tumor growth and angiogenesis and decreasing apoptosis and immune surveillance [42, 43]. For example, cortisol inhibits apoptosis, reduces p53 functions, and induces cell cycle arrest that, in turn, promotes tumor growth and therapy resistance [44–46]. Moreover, adrenaline and noradrenaline stimulate the EMT process, angiogenesis, anoikis (resistance to apoptosis), and tumor cell invasion [43, 47, 48].

Our study showed increased cortisol, adrenaline, and noradrenaline concentrations in the blood from LAC patients compared to healthy subjects. The rise of these hormones' concentrations can be associated with the stress generated when patients face the cancer diagnosis, worry about treatment side effects, question whether therapy will cure the disease, confront the imminent death, and are concerned about the cost of treatment when they have a low economic income. In the case of tobacco smoker cancer patients, they are advised to quit smoking, and therefore, they also experience stress since smoking is a way to deal with situations they cannot control [49, 50]. Additionally, these patients may feel guilty since they recognize that tobacco is the cause of their disease. All these psychological events may explain the increase in cortisol and adrenaline observed in tobacco smokers. In addition, nitrosamine 4-(methylnitrosamino)-1-(3-pyridyl)-1-butanone (NNK), a carcinogen derived from nicotine, is an agonist of the *α*7-nicotinic acetylcholine receptor (nAChR) that, when activated, favors the release of adrenaline and noradrenaline inducing cancer progression through their interaction with the *β*-adrenergic receptors (*β*-ARs) [51]. Moreover, NNK has a higher affinity for the *β*-ARs than adrenaline and noradrenaline. NKK also binds to the *α*4nAChR blocking *γ*-aminobutyric acid (GABA) release that contributes to the inhibition of cancer cell proliferation and migration [51].

Because chronic stress has been considered a factor involved in cancer onset and evolution, and MMPs have an important role in cancer progression, a correlation between an increase in stress hormones and MMPs synthesis has been explored. For example, MMP-9 expression was stimulated by cortisol and noradrenaline in macrophages from ovarian cancer patients [52]. Furthermore, noradrenaline can increase MMP-2, MMP-9, and VEGF synthesis and the metastatic potential of pancreatic cancer cells [53]. Similar results were obtained in a chronic stress model of oral cancer in mice, where cortisol and catecholamines augmented MMP-2 and VEGF expression in the tumor tissue [54]. According to these findings, the correlation between stress hormones' concentrations and MMPs/TIMPs levels in blood samples may indicate the degree of cancer progression using a noninvasive IVD test. In this context, positive correlations between serum MMP-9 and baseline morning cortisol levels and between serum MMP-9 and cortisol concentrations after administration of dexamethasone were observed in patients with functioning adrenal tumors [55].

Our work analyzed correlations between blood concentrations of stress hormones and MMPs/TIMPs in control and lung adenocarcinoma subjects. In this regard, when cortisol concentrations increased, MMP-12 levels decreased (*r*_*s*_ = −0.334), while TIMP-2 levels augmented (*r*_*s*_ = 0.239) in the healthy subjects. Perhaps, although cortisol levels will be elevated in healthy subjects, MMP-12 concentrations would remain low in the absence of a stimulus such as neoplastic cell proliferation and hypoxia, which are TME conditions involved in angiogenesis during cancer's early stages [3]. Likewise, TIMP-2 increased levels may participate in the control of MMPs enzymatic activity. On the contrary, a significant correlation between cortisol and MMP-12 was not observed in LAC patients, but increased cortisol levels were associated with high TIMP-2 (*r*_*s*_ = 0.409) and TIMP-1 (*r*_*s*_ = 0.392) concentrations, particularly in patients exposed to wood smoke. Both TIMPs are involved in proMMPs' activation processes (see above). In addition, a rise in adrenaline was associated with a decrease in MMP-2 (*r*_*s*_ = −0.256), and an increase in noradrenaline correlated with high MMP-2 levels (*r*_*s*_ = 0.322) in healthy subjects. Therefore, both catecholamines might be participating, at least in part, in the mechanisms that keep blood MMP-2 levels in balance in this group. Regarding the association between adrenaline and MMPs/TIMPs blood levels in LAC patients, a weak correlation was found between adrenaline and MMP-9 (*r*_*s*_ = 0.197). Likewise, augmented noradrenaline concentrations were weakly associated with a rise in MMP-2 levels in the N group (*r*_*s*_ = 0.391). Additionally, noradrenaline augmented levels correlated with high levels of MMP-9 (*r*_*s*_ = 0.332) in the control group but not in cancer patients. This correlation may be associated with other yet undefined conditions different from cancer. In general terms, few correlations between MMPs/TIMPs and stress hormones concentrations in blood were observed, and these correlations were weak in LAC patients. Clearly, further research in vitro and in vivo in lung cancer models is needed to establish if stress hormones have an effect on MMPs' and TIMPs' expression. Additionally, it would be valuable to investigate whether this effect can be determined by more sensitive techniques that could allow its measurements to be considered a reliable marker of cancer progression.

## 5. Conclusions

Differences in blood concentrations of MMPs and TIMPs among lung adenocarcinoma and healthy subjects were identified, and therefore, the levels of these molecules might have potential therapeutic value if used as prognostic markers of disease aggressiveness. In addition, cortisol and catecholamine levels were increased in cancer patients, but their increment differed between risk factor groups. Although the stress hormones' effects on lung cancer progression are still controversial, the psychological approach of lung cancer patients with advanced stages of the disease and the use of *β*-blockers to interfere with catecholamine interactions with their receptors may improve disease prognosis and patients' quality of life.

Likewise, it would have been very interesting to compare the data obtained in the present work with those from healthy subjects with exposure to wood or tobacco smoke and healthy tobacco smokers; however, healthy individuals lacking symptoms do not attend the hospital, and therefore, such data are difficult to obtain.

Finally, MMPs and TIMPs, as well as stress hormones, contribute to lung cancer progression, although in this study it was not possible to establish a well-defined correlation among them.

## Figures and Tables

**Table 1:** Lung adenocarcinoma patients' clinical characteristics.

Characteristics	All patients	W	N	TS	PS	TW
Subjects	104	36.5 (38/104)	25 (26/104)	21.2 (22/104)	9.6 (10/104)	7.7 (8/104)
Gender						
Women	45.2 (47/104)	51.1 (24/47)	25.5 (12/47)	2.1 (1/47)	14.9 (7/47)	6.4 (3/47)
Age	57.7 ± 11.9	62.3 ± 11.5	49.9 ± 11.5	70	54.4 ± 8.3	56.3 ± 6.8
Men	54.8 (57/104)	24.6 (14/57)	24.6 (14/57)	36.8 (21/57)	5.3 (3/57)	8.7 (5/57)
Age	61.9 ± 10.7	62.1 ± 11.1	58.9 ± 14.6	63.9 ± 8.2	58 ± 6.2	64.6 ± 8.9
Stage (TNM)						
IIA	0.9 (1/104)	—	—	—	10 (1/10)	—
IIIA	2.9 (3/104)	—	11.5 (3/26)	—	—	—
IIIB	5.8 (6/104)	5.3 (2/38)	7.7 (2/26)	9.1 (2/22)	—	—
IV	90.4 (94/104)	94.7 (36/38)	80.8 (21/26)	90.9 (20/22)	90 (9/10)	100 (8/8)

Data are shown as a % (number of patients/total patients). Age is expressed in years as mean ± SD. LAC: lung adenocarcinoma; W: LAC associated with wood smoke exposure; N: LAC patients with no recognizable risk factor; TS: LAC associated with tobacco smoking; PS: LAC associated with passive smoking; TW: LAC associated with tobacco smoking and wood smoke exposure.

**Table 2:** CEA plasma concentrations.

Group	*n*	CEA ng/ml	Range ng/ml
LAC	70	62.5 ± 14.9	0.5-626.43
W	29	49.5 ± 16.9	0.94-459.68
N	14	99.1 ± 45.7	0.5–495.63
TS	13	39.7 ± 15.3	1.08-160
PS	7	13.1 ± 10.5	0.64–75.78
TW	7	135.4 ± 89.3	0.62–626.43
<5 ng/ml	30	2.3 ± 0.3	0.5–4.92
>5 ng/ml	40	107.7 ± 23.8	6.01–626.43

Data are expressed in mean ± SE. CEA: carcinoembryonic antigen; LAC: lung adenocarcinoma; W: LAC associated with wood smoke exposure; N: LAC patients with no recognizable risk factor; TS: LAC associated with tobacco smoking; PS: LAC associated with passive smoking; TW: LAC associated with tobacco smoking and wood smoke exposure.

## Data Availability

The data used during the present study are available from the corresponding author upon reasonable request.
